# Automatic Coronary Artery Calcium Scoring on Radiotherapy Planning CT Scans of Breast Cancer Patients: Reproducibility and Association with Traditional Cardiovascular Risk Factors

**DOI:** 10.1371/journal.pone.0167925

**Published:** 2016-12-09

**Authors:** Sofie A. M. Gernaat, Ivana Išgum, Bob D. de Vos, Richard A. P. Takx, Danny A. Young-Afat, Noor Rijnberg, Diederick E. Grobbee, Yolanda van der Graaf, Pim A. de Jong, Tim Leiner, Desiree H. J. van den Bongard, Jean-Philippe Pignol, Helena M. Verkooijen

**Affiliations:** 1 Department of Epidemiology, Julius Center, University Medical Center Utrecht, Utrecht, The Netherlands; 2 Image Sciences Institute, University Medical Center Utrecht, Utrecht, The Netherlands; 3 Department of Radiology, University Medical Center Utrecht, Utrecht, The Netherlands; 4 Devision of Internal Medicine, Academic Medical Center, Amsterdam, The Netherlands; 5 Department of Radiation Oncology, University Medical Center Utrecht, Utrecht, The Netherlands; 6 Department of Radiation Oncology, Erasmus Medical Center, Rotterdam, The Netherlands; 7 Imaging Division, University Medical Center Utrecht, Utrecht, The Netherlands; Universitatsklinikum Freiburg, GERMANY

## Abstract

**Objectives:**

Coronary artery calcium (CAC) is a strong and independent predictor of cardiovascular disease (CVD) risk. This study assesses reproducibility of automatic CAC scoring on radiotherapy planning computed tomography (CT) scans of breast cancer patients, and examines its association with traditional cardiovascular risk factors.

**Methods:**

This study included 561 breast cancer patients undergoing radiotherapy between 2013 and 2015. CAC was automatically scored with an algorithm using supervised pattern recognition, expressed as Agatston scores and categorized into five categories (0, 1–10, 11–100, 101–400, >400). Reproducibility between automatic and manual expert scoring was assessed in 79 patients with automatically determined CAC above zero and 84 randomly selected patients without automatically determined CAC. Interscan reproducibility of automatic scoring was assessed in 294 patients having received two scans (82% on the same day). Association between CAC and CVD risk factors was assessed in 36 patients with CAC scores >100, 72 randomly selected patients with scores 1–100, and 72 randomly selected patients without CAC. Reliability was assessed with linearly weighted kappa and agreement with proportional agreement.

**Results:**

134 out of 561 (24%) patients had a CAC score above zero. Reliability of CVD risk categorization between automatic and manual scoring was 0.80 (95% Confidence Interval (CI): 0.74–0.87), and slightly higher for scans with breath-hold. Agreement was 0.79 (95% CI: 0.72–0.85). Interscan reliability was 0.61 (95% CI: 0.50–0.72) with an agreement of 0.84 (95% CI: 0.80–0.89). Ten out of 36 (27.8%) patients with CAC scores above 100 did not have other cardiovascular risk factors.

**Conclusions:**

Automatic CAC scoring on radiotherapy planning CT scans is a reliable method to assess CVD risk based on Agatston scores. One in four breast cancer patients planned for radiotherapy have elevated CAC score. One in three patients with high CAC scores don't have other CVD risk factors and wouldn't have been identified as high risk.

## Introduction

Breast cancer patients treated with adjuvant treatments such as radiotherapy or chemotherapy may be at increased absolute risk of treatment-induced cardiotoxicity[1–4]. This risk is higher in patients with pre-existing cardiovascular disease (CVD) risk factors[5,6]. One of the strongest individual predictive factors of CVD risk is the presence and amount of coronary artery calcium (CAC), representing the extent of coronary atherosclerosis, independent of traditional CVD risk factors like hypercholesterolemia, hypertension or diabetes[7]. The amount of CAC is most commonly expressed as Agatston score, and categorized Agatston scores are clinically used to express the risk of CVD events[8]. Asymptomatic individuals with Agatston scores of 100 and higher, and without other CVD risk factors, have a 20% 10-year risk of a CVD event, compared to 1% in asymptomatic individuals without CAC[8,9].

CAC is quantified in the main coronary arteries, namely left main (LM), left anterior descending (LAD), left circumflex (LCX) and right coronary artery (RCA). Standardly, CAC is quantified on cardiac computed tomography (CT) scans that are made using ECG-triggering minimizing cardiac motion and thus enabling good visualization of the CAC. Nevertheless, CAC can also be quantified using any CT scans visualizing the heart, and previous studies have shown that CAC scores determined using non-dedicated acquisition protocols, i.e. without ECG-synchronization and using low radiation dose, are predictive of future CVD events[10–15]. In clinic, CAC scoring is performed by manual expert annotation, which is time-consuming and tedious when performed using non-dedicated CT scans due to presence of artefacts caused by cardiac motion, high noise levels caused by lower radiation dose and partial volume effect caused by decreased image resolution[16,17]. To overcome this and enable large scale studies, several algorithms for automatic CAC scoring in both dedicated cardiac, and non-dedicated chest CT scans have been proposed[18–23].

All breast cancer patients treated with radiotherapy routinely undergo low-dose planning CT scans of the chest. As the coronary arteries are visualized on these scans, CAC can be quantified without exposing patients to additional radiation and without additional costs. However, it is unknown whether radiotherapy planning CT scans of breast cancer patients can reliably be used for (automatic) CAC scoring.

The objective of this study was to evaluate reproducibility of automatic CAC scoring on breast radiotherapy planning CT scans and to examine the association between CAC scores and traditional CVD risk factors.

## Methods and Materials

### Study design and patients

This study was conducted within the prospective Utrecht cohort for Multiple BReast cancer intErvention studies and Long-term evaLuAtion (UMBRELLA). The UMBRELLA cohort was approved by the Medical Ethics Review Committee of the University Medical Center Utrecht (UMBRELLA protocol number = 15–165). Recruitment in the cohort started in October 2013 and all breast cancer patients planned for radiotherapy were eligible for participation. Until March 2015, 628 consecutive breast cancer patients signed informed consent of the UMBRELLA study and were enrolled. Six patients withdrew informed consent, 60 patients did not undergo a planning CT scan, and one patient was excluded due to CT image artifacts caused by metal implants, leaving 561 patients for inclusion.

Patient and treatment characteristics, e.g. age at time of CT scan, tumor stage at diagnosis according to the International Union against Cancer (UICC) classification of malignant tumors (TNM)[24] and type of treatments, were systematically collected within the context of the UMBRELLA cohort and based on clinical records and national cancer registry data. Traditional CVD risk factors, including diabetes, hypertension, hypercholesterolemia, smoking status and history of CVD, were extracted from electronic medical files at the radiotherapy department. As for diabetes, hypertension, hypercholesterolemia, smoking status and history of CVD, patients were scored as positive when medication had been prescribed or when it had been explicitly noted in the electronic files. Smoking status was categorized as never or not reported, former or current. History of CVD was scored as positive in case patients had experienced ischaemic heart disease, heart failure, stroke, atrial fibrillation or angina pectoris before start of the radiotherapy.

### Procedures

Radiotherapy planning CT scans were performed with a Brilliance CT (Philips Medical Systems) scanner with 16 x 0.75 mm collimation, 120 kVp, 3 mm section thickness, without contrast enhancement, without ECG-synchronization. All patients underwent a planning CT scan without breath-hold, and patients with left-sided breast cancer underwent an additional planning CT scan with breath-hold.

Automatic CAC scoring was performed in all patients to assess presence and the amount of CAC. CAC was automatically scored in the LM, LAD, LCX and RCA with the algorithm described by Isgum et al[23]. Briefly, CAC was identified using a supervised machine learning approach. Following clinical procedure, three-dimensional connected components above the standard threshold of 130 Hounsfield Units (HU) were considered candidate calcifications. Based on their volume, spatial and texture characteristics, CAC was identified using supervised classification and expressed as Agatston scores, volume (mm³) and number of CAC[8]. The scan with the highest Agatston score was selected for patients with multiple CT scans. Scans with automatically determined CAC scores of 1000 and above (n = 6) were manually inspected and corrected if needed. Each patient was assigned to one of five CVD risk categories based on Agatston score: low (0), fair (1–10), moderate (11–100), intermediate (101–400), high (> 400)[17,25,26].

In the current study, we assessed (1) reproducibility between automatic and manual expert scoring, (2) interscan reproducibility of automatic CAC scoring, and (3) associations between CAC scores and other traditional CVD risk factors.

Automatic and manual CAC scores were compared in 163 patients. Manual scoring was performed in the first 79 consecutive patients with automatically determined CAC scores above 0 and in 84 randomly selected patients without CAC. CAC was manually annotated by a radiologist in training with experience in over 1000 scans, who was blinded to the automatically determined CAC scores and patient’s characteristics, except for date of birth.

Interscan reproducibility of automatic CAC scoring was assessed in all 294 patients having received (at least) two CT scans, either on the same day (82%) or within a maximum of five months (18%)[27].

Associations between CAC scores and traditional CVD risk factors were assessed in all 36 patients with automatic CAC scores above 100, 72 randomly selected patients with scores 1–100, and 72 randomly selected patients without CAC.

### Statistical analysis

Demographics, tumor characteristics, treatment details and CAC scores were described for all patients. Reproducibility between automatic and manual CAC scoring as well as the interscan reproducibility of automatic CAC scoring was assessed with reliability and agreement analyses[28]. Reliability—agreement beyond chance—of CAC score categories was assessed with Cohen’s linearly weighted kappa (κ)[29]. Reliability of continuous CAC score was measured with Intraclass Correlation Coefficient (ICC). The two-way random effects and absolute agreement ICC was used to assess reliability between automatic and manual CAC scoring, taking into account the variance between patients and structural differences between automatic and manual CAC scoring. The two-way random consistency ICC was used to assess reliability between two automatically scored scans. Agreement—degree to which CAC scores are identical between methods (i.e. automatic versus manual CAC scoring and automatic versus automatic CAC scoring)—of CAC score categories was assessed with proportional agreement. Agreement of continuous CAC score was assessed with Bland-Altman plots and its back log transformed 95% limits of agreement due to inconsistent variances, which increase with higher CAC scores.

Overall associations between CAC scores and traditional CVD risk factors were assessed with Chi-Square and Kruskal-Walles tests for categorical and continuous variables respectively.

Analyses were performed with IMB SPSS statistics version 20 and an online statistical tool (http://vassarstats.net/kappa.html).

## Results

Median age at time of CT scan of all 561 breast cancer patients in the present study was 61 years (interquartile range: 54–68), and 355 (63%) patients were diagnosed with Stage 1 disease (Table 1). Almost all patients were treated with surgery and radiotherapy (n = 556, 99%), and 427 (76%) patients had a CAC score of zero. Of the 134 (24%) patients with a CAC score above zero, 36 (27%) patients had a score above 100. Six CT scans had an automatically determined CAC score of 1000 and above, and these high CAC scores were caused by large CAC depositions in the mitral annulus. Three of those were corrected to a CAC score of zero, and two were corrected to a score between 50 and 100. One scan was corrected to a CAC score above 2000.

**Table 1:** Demographics, tumor and treatment characteristics, and CAC (Agatston) scores of 561 breast cancer patients.

	n (%)
**Median age at time of scan in years (interquartile range)**	61 (54–68)
**Tumor stage at diagnosis**	
In situ	65 (11)
1	354 (63)
2	118 (21)
3	21 (4)
4	3 (1)
**Combination of treatments**	
Surgery + RT	216 (39)
Surgery + RT + CT	69 (12)
Surgery + RT + HT	101 (18)
Surgery + RT + CT + HT	170 (30)
Other ^a^	5 (1)
**Median CAC in Agatston score (interquartile range)**	3 (0–55)
**CAC in Agatston score categories**	
0	427 (76)
1–10	46 (8)
11–100	52 (9)
101–400	28 (5)
> 400	8 (2)
**Median volume of CAC in mm³ (interquartile range)**	7 (0–86)
**Median number of CAC (interquartile range)**	1 (0–2)

Abbreviations: CAC = coronary artery calcification; RT = radiotherapy; CT = chemotherapy; HT = hormonal treatment; mm³ = cubic millimeter

^a^ No surgery + CT + HT and/ or RT, only surgery, or surgery with CT or HT

### Automatic versus manual CAC scoring

**Fig 1:**
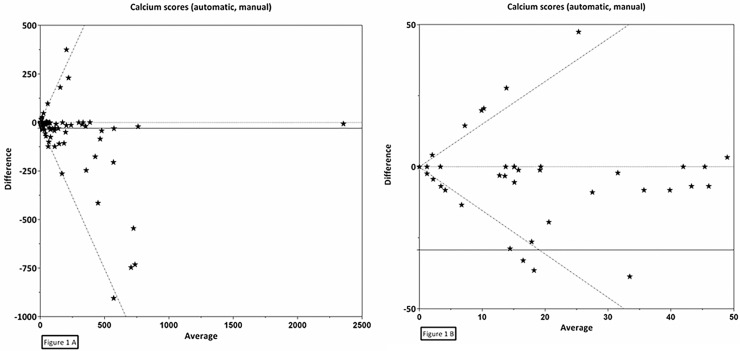
Bland-Altman plot for the agreement between automatically and manually determined CAC on planning breast CT. CAC (Agatston) scores were assessed automatically and manually of 163 breast cancer patients using radiotherapy planning CT scans. Mean (X) = -29.3, standard deviation = 131.2, back log transformed upper limit of agreement = 1.5*X, back log transformed lower limit of agreement = -1.5*X, 1A is full plot, 1B is zoomed plot.

Reproducibility between automatic and manual CAC scoring was assessed in 163 patients, including 58 scans performed with breath-hold and 105 without breath-hold. The reliability of CAC score categories (κ) was 0.80, 95% Confidence Interval (CI): 0.74–0.87, and slightly higher for scans performed with breath-hold (κ = 0.86, 95% CI: 0.77–0.96) than for those without breath-hold (κ = 0.77, 95% CI: 0.68–0.85) (Tables 2 and 3). The proportion of agreement for CVD risk categories was also high at 0.79 (95% CI: 0.72–0.85), and higher for scans performed with breath-hold (0.88, 95% CI: 0.76–0.95) than for those without breath-hold (0.74, 95% CI: 0.65–0.82). The reliability of continuous CAC score (ICC) was 0.86, 95% CI: 0.81–0.89, and higher for scans performed with breath-hold (ICC = 0.95, 95% CI: 0.91–0.97) than for those without breath-hold (ICC = 0.66, 95% CI: 0.54–0.76) (Table 3). For continuous CAC scores a Bland-Altman plot showed a mean difference between the automatic and manual scored scans of -29.3 with back log transformed 95% limits of agreement as a function of the average (X) of -1.5X and 1.5X (Fig 1A and 1B).

**Table 2:** Agreement between automatically and manually determined Agatston scores on 163 breast planning CT scans.

Manual coronary artery calcium in Agatston score categories	Automatic coronary artery calcium in Agatston score categories					
	0	1–10	11–100	101–400	> 400	Total
**0**	75	1	5	0	0	81
**1–10**	4	2	1	1	0	8
**11–100**	4	2	31	2	0	39
**101–400**	1	0	7	14	0	22
**> 400**	0	0	0	6	7	13
**Total**	84	5	44	23	7	163

**Table 3:** Reproducibility of automatic calcium scoring versus manual, and interscan reproducibility of automatic scoring, on breast planning CT.

	Categorical ^a^		Continuous
	Linearly weighted kappa (95% CI)	Proportion of agreement (95% CI)	Intraclass correlation coefficient of calcium (Agatston) scores (95% CI)
**Automatic vs. manual (n = 163)**	0.80 (0.74–0.87)	0.79 (0.72–0.85)	0.86 (0.81–0.89)
Breath-hold (n = 58)	0.86 (0.77–0.96)	0.88 (0.76–0.95)	0.95 (0.91–0.97)
Without breath-hold (n = 105)	0.77 (0.68–0.85)	0.74 (0.65–0.82)	0.66 (0.54–0.76)
**Automatic vs. automatic(n = 294)**	0.61 (0.50–0.72)	0.84 (0.80–0.89)	0.34 (0.23–0.44)

Abbreviations: CAC = coronary artery calcium; CI = Confidence Interval

^a^ Cardiovascular risk categories of coronary artery calcium based on Agatston score: 0, 1–10, 11–100, 101–400, > 400

### Interscan reproducibility of automatic CAC scoring

**Fig 2:**
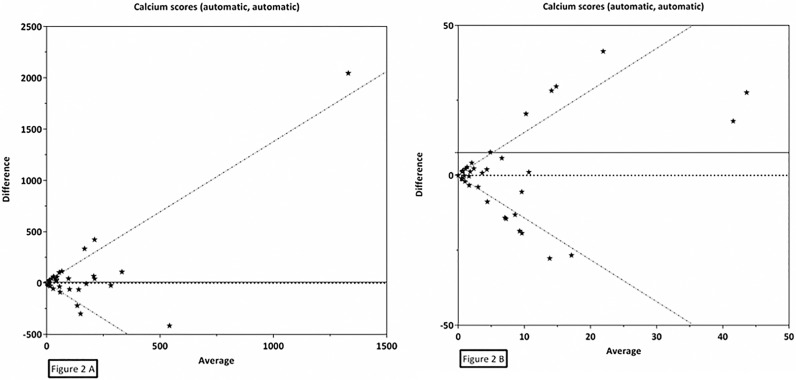
Bland-Altman plot for the agreement of automatically determined CAC on planning breast CT. CAC (Agatston) scores were assessed in two scans of the same patient in a set of 294 breast cancer patients using radiotherapy planning CT scans. Mean (X) = 7.6, standard deviation = 128.7, back log transformed upper limit of agreement = 1.4*X, back log transformed lower limit of agreement = -1.4*X, 2A is full plot, 2B is zoomed plot.

Interscan reproducibility of automatic CAC scoring was assessed in all 294 patients who underwent two CT scans: 237 (81%) patients underwent one CT scan performed with breath-hold and one without, 50 (17%) underwent two scans performed without breath-hold and 7 (2%) underwent two scans performed with breath-hold. Reliability of CVD risk categories (κ) was 0.61, 95% CI: 0.50–0.72, and the proportion of agreement for CVD risk categories was 0.84, 95% CI: 0.80–0.89 (Tables 3 and 4). Reliability of continuous CAC score (ICC) was 0.34, 95% CI: 0.23–0.44 (Table 3). For continuous CAC scores a Bland-Altman plot showed a mean difference between the two automatically scored scans of 8.6 with back log transformed 95% limits of agreement as a function of the average (X) of -1.4X and 1.4X (Fig 2A and 2B).

**Table 4:** Agreement of automatically determined Agatston scores on radiotherapy planning CT of 294 breast cancer patients.

Automatic coronary artery calcium in Agatston score categories	Automatic coronary artery calcium in Agatston score categories					
	0	1–10	11–100	101–400	> 400	Total
**0**	228	10	6	1	0	245
**1–10**	8	9	4	0	0	21
**11–100**	4	2	6	3	0	15
**101–400**	1	2	1	6	1	11
**> 400**	1^a^	0	0	1	0	2
**Total**	242	23	17	11	1	294

^**a**^ Patient underwent one CT scan with breath-hold and one without breath-hold. The scan with breath-hold had an automatic coronary artery calcium score of 423, which was in agreement with the manual coronary artery calcium score after inspection. The scan without breath-hold had an automatic coronary artery calcium score of zero, which was manually inspected and corrected to a score of 885. The disagreement is caused by missed coronary artery calcium in the left anterior descending artery.

### Associations between categorized CAC scores and traditional CVD risk factors

Diabetes was significantly more prevalent among patients with CAC scores above 100 than in those with CAC scores of zero: 27.8% versus 5.6% (p = 0.001) (Table 5). Patients with CAC scores above 100 had more often three to five CVD risk factors compared to patients with scores between 1–100 or with CAC scores of zero: 33.3%, 16.7%, and 9.7% respectively (p = 0.023). Interestingly, ten of the 36 patients (27.8%) with CAC scores above 100 did not have any other traditional CVD risk factor and would have been missed evaluating the risk clinically.

**Table 5:** Cardiovascular risk factors in relation to calcium (Agatston) scores of 108 breast cancer patients.

	CAC score: 0	CAC score: 1–100	CAC score: > 100	p value
	n = 72 (%)	n = 72 (%)	n = 36 (%)	
**Median CAC (Agatston) score (interquartile range)**	0 (0–0)	12 (3–30)	257 (134–389)	<0.001
**Median age at time of scan in years (interquartile range)**	57 (50–64)	62 (55–67)	70 (63–74)	<0.001
**Diabetes**				0.001
Yes	4 (5.6)	5 (6.9)	10 (27.8)	
No	68 (94.4)	67 (93.1)	26 (72.2)	
**Hypertension**				0.007
Yes	15 (20.8)	31 (43.1)	16 (44.4)	
No	57 (79.2)	41 (56.9)	20 (55.6)	
**Hypercholesterolemia**				0.492
Yes	9 (12.5)	14 (19.4)	5 (13.9)	
No	63 (87.5)	58 (80.6)	31 (86.1)	
**Smoking status**				0.437
Current	8 (11.1)	12 (16.7)	3 (8.3)	
Former	11 (15.3)	15 (20.8)	10 (27.8)	
Never/ not reported	53 (73.6)	45 (62.5)	23 (63.9)	
**History of CVD**				0.019
Yes	15 (20.8)	13 (18.1)	15 (41.7)	
No	57 (79.2)	59 (81.9)	21 (58.3)	
**Number of CVD risk factors**				0.023
0	34 (47.3)	20 (27.7)	10 (27.8)	
1	16 (22.2)	19 (26.4)	8 22.2)	
2	15 (20.8)	21 (29.2)	6 (16.7)	
3–5	7 (9.7)	12 (16.7)	12 (33.3)	

Abbreviations: CAC = coronary artery calcium, CVD = cardiovascular disease

## Discussion

This study shows that automatic CAC scoring on radiotherapy planning CT scans is a reliable method to assess CVD risk categories based on CAC scores. One in four breast cancer patients planned for radiotherapy have elevated CAC score. In a small study of breast cancer patients, one in three patients with high CAC do not have any other CVD risk factor and may hence be missed in the cardiac morbidity risk evaluation.

The algorithm to automatically score CAC is developed for low-dose, non-dedicated CT scans acquired in a lung cancer screening trial[23]. In this context, Takx et al. evaluated reproducibility of the algorithm in 1749 participants by comparing it to manual scoring by a radiologist[17]. This study showed a very good reliability between automatic and manual CAC scoring, with a κ of 0.85 for CVD risk categorization and ICC of 0.90 for continuous CAC score. Our study shows comparable, albeit slightly lower, reliability results for automatic versus manual CAC scoring, with a κ = 0.80 for CVD risk categorization and ICC of 0.86 for continuous CAC score. This is not surprising since the algorithm was trained with non-representative training data, namely low-dose chest CT scans[23]. Retraining the algorithm with representative radiotherapy planning CT scans of breast cancer patients will most likely increase its performance.

In this study, CT scans with an automatically determined CAC score of 1000 and higher were inspected. Five scans contained large false positives representing CAC in the mitral annulus that were strongly affected by cardiac motion and difficult to differentiate from CAC in LCX in non-dedicated CT scans[30]. Please note that such calcifications are also predictive of future CVD events[31]. Reproducibility between automatic and manual CAC scoring was much higher in CT scans performed with breath-hold than in those without. Breath-holding technique is often used for patients who receive left-sided radiotherapy in order to minimize heart radiation exposure[32]. CT scans with breath-hold show reduced respiration motion artifacts allowing for more accurate automatic CAC scoring, and enhances reproducibility between automatic and manual CAC scoring. The interscan reliability of CVD risk categories based on CAC scores between two automatically scored scans was much lower than the reliability between automatic and manual CAC scoring (0.61 versus 0.80, respectively). Difference in respiratory motion artifacts between CT scans performed with and without breath-hold has very likely contributed to this lower reliability of automatic CAC scoring, since 237 out of 294 (81%) patients had one CT scan performed with breath-hold and one scan without. Around 50% of all breast cancer patients are treated with radiotherapy and therefore routinely undergo planning CT scans[32,33].

Previous studies have shown that CAC is a stronger risk factor than traditional CVD risk factors, such as diabetes, hypertension and smoking status[34–36]. CAC scores of 100 and above are related to an increased risk of multivessel disease, coronary heart disease and overall CVD events[9,35,37]. In our study, 10 out of 36 patients (27.8%) with CAC scores above 100 did not have any other CVD risk factor. Though these patients are at high CVD risk, they would not have been detected as high risk based on traditional CVD risk factors only.

We acknowledge that this study has limitations. Information on traditional CVD risk factors of breast cancer patients were retrieved from medical files at the radiotherapy department. These files are filled out by radiation oncologists or oncology nurses and may have resulted in underreporting of smoking and other traditional CVD risk factors. Moreover, we are not able to provide a cardiovascular risk score as blood pressure and cholesterol levels, which are necessary for, are not routinely measured in clinic. Another limitation is that we cannot assume an association between the presence and amount of CAC measured on non-dedicated radiotherapy planning CT scans and increased CVD risk. The Multi-Ethnic Study of Atherosclerosis (MESA) showed a strong association between the presence and amount of CAC and increased CVD risk. However, MESA measured CAC on dedicated cardiac CT scans and included a different study population as our study with different ethnicities (white, black, Hispanic, Asian), males and females, and without active cancer treatment[9,34,36]. Moreover, presence and amount of CAC have shown to be predictive in distinguishing patients with increased CVD risk based on CAC scores using non-dedicated chest CT scans of subjects in lung cancer screening trials[12,38,39].

Furthermore, so far there are no treatments to slow down or arrest the progression of CAC, and trial results have to be waited for. A randomized placebo-controlled trial is investigating the effect of 24-month treatment with menaquinon-7 supplementation (vitamin K antagonist) on the progression of CAC[40]. Moreover, a Dutch randomized-controlled trial is investigating whether early detection of CVD risk based on CAC score with subsequent lifestyle and/ or treatment intervention will reduce CVD morbidity and mortality in a high-risk population[41].

## Conclusions

In conclusion, automatic CAC scoring on radiotherapy planning CT scans is a reliable method to assess CVD risk categories based on CAC scores, preferably at breath-hold examinations, without additional radiation exposure or costs involved. In this prospective cohort study of 561 patients, we demonstrated that one in four patients has elevated CAC, and that one in three patients with high CAC scores don’t have other CVD risk factors and would therefore not have been identified as high risk.

Knowing a patient’s baseline CVD risk is essential when evaluating a left-sided radiotherapy planning CT scan, given the dose received by the heart during radiotherapy is associated with an increased risk of major CVD events[42]. The clinical relevance of automatic CAC scoring on planning CT scans in relation to increased absolute risk of a major CVD event still needs to be evaluated. The future clinical application of the presence and amount of CAC measured on planning CT scans, and the patient’s corresponding CVD risk, may be twofold. Radiation and medical oncologists may use it to identify patients who are candidates for less cardiotoxic treatments, and may refer patients with high cardiac morbidity to cardiologists for further diagnostic evaluation and treatment. General practitioners may use the information to start lifestyle interventions and/or treatments such as antihypertensives, to reduce the patient’s CVD risk.

In a follow-up study, the automatic CAC scoring software will be adapted and optimized for radiotherapy planning CT scans of breast cancer patients. Moreover, associations between CAC assessed on radiotherapy planning CT scans and CVD risk (factors) of breast cancer patients will be investigated including patient’s preferences and needs regarding disclosure of their CAC scores.
